# Asphalt Mixes Processed with Recycled Concrete Aggregate (RCA) as Partial Replacement of the Natural Aggregate

**DOI:** 10.3390/ma14154196

**Published:** 2021-07-27

**Authors:** Carlos U. Espino-Gonzalez, Wilfrido Martinez-Molina, Elia M. Alonso-Guzman, Hugo L. Chavez-Garcia, Mauricio Arreola-Sanchez, Adria Sanchez-Calvillo, Marco A. Navarrete-Seras, Jorge A. Borrego-Perez, Juan F. Mendoza-Sanchez

**Affiliations:** 1 Faculty of Civil Engineering, Universidad Michoacana San Nicolas de Hidalgo, Morelia 58070, Mexico; carlos.espino@umich.mx (C.U.E.-G.); elia.alonso@umich.mx (E.M.A.-G.); luis.chavez@umich.mx (H.L.C.-G.); mauricio.arreola@umich.mx (M.A.-S.); mnavarrete@umich.mx (M.A.N.-S.); jorge.borrego@umich.mx (J.A.B.-P.); 2 Faculty of Architecture, Universidad Michoacana San Nicolás de Hidalgo, Morelia 58070, Mexico; adria.sanchez.9@hotmail.com; 3 Mexican Institute of Transportation (IMT), Queretaro 76703, Mexico; fmendoza@imt.mx

**Keywords:** asphalt mix, recycled concrete, petrous aggregates, environmental saving, Marshall methodology, RCA

## Abstract

Materials play a fundamental role in any branch of civil engineering. From ancient times to the present day, society has required enormous amounts of construction materials, which implies an excessive exploitation of the natural environment. The present research work consisted in the design and development of asphalt mixes with a partial substitution of the natural aggregate (NA) by means of recycled concrete aggregate (RCA). The mix was designed with the Marshall methodology, considering the next percentages of substitution and addition by mass: 90% NA and 10% RCA; 80% NA and 20% RCA; 70% NA and 30% RCA. The mixtures were elaborated and analysed under the international standards and the Mexican regulation of the Communications and Transport Ministry, to determine the best option regarding their performance. The materials were characterized according to the current regulations and later employed in the mixes design. A total of 38 specimens were elaborated for each mixture, determining the optimum asphalt content; after that, mechanical tests were performed to analyse and determine the best results. In the aftermath of the examination of all mixtures, we concluded that the 70%AN/30%RCA is the best alternative option according to its performance and numeric results, complying with the cited regulations, and allowing a lower content of asphalt during the process.

## 1. Introduction

One of the biggest challenges of the construction industry is the integration of low-embodied energy materials, for this reason the tendency to reuse waste materials has increased during the last years. Within this framework, these solid wastes have been used in building purposes, while it is not clear if they comply with the physical and mechanical requirements demanded; nevertheless, they are used to diminish the environmental impact which the conventional prime materials imply [1,2]. To reduce the intake of natural resources, the utilization of construction generated wastes, mainly concrete and ceramic materials, has been promoted, incorporating them to the new designed mixtures substituting partially or entirely the petrous aggregates [3].

Notwithstanding, the specific nature and origin of these materials provoke very different physical, chemical and mechanical properties than the natural petrous aggregates, which modify the behaviour and qualities of the asphalt mix. The construction industry generates approximately 900 million tonnes of building and demolition wastes (CDW) [4]; in the year 2015, 12 million tonnes were reported only in Mexico [5]. The Mexican Chamber of Construction Industry (CMIC) estimates that only the 4% of CDW are exploited: the 3% in recycling processes and the other 1% in building reuse. Only Mexico City produces 12,000 tonnes per day of solid urban waste, and 50% of these (6000 tonnes per day), are CDW [6]. In the large majority of countries, the waste disposal of CDW is not extended, causing their dropping into public roads, clandestine garbage dumps or the ends of roadways and highways, polluting the environment and scenery and wasting the remaining service life of these materials.

CDW are composed by many materials utilised in construction purposes, the main ones (from 65 to 75%) being stone type: concrete, mortars, ceramic bricks, tiles, slabs, etc. The composition of these materials makes them valuable and suitable to continue with the construction life cycle.

Due to the big environmental impact of the construction industry and the great generation of CDW across the world, and regarding the lack of procedures and regulations to exploit them, it should be a must to design and incorporate new management plans and regulations to help reducing the natural resources exploitation, the total waste volume, diminishing sanitary landfills and CO_2_ emissions, strengthening the recycling industry and generating a saving in the transportation costs. The environmental objectives for the reuse, recycle and valorisation of the CDW concentrate on their management, being the main goal the reutilization of these materials for new processes. It is essential to integrate the separate collection as one of the first phases and next initiate the transformation process of the residues to recycled construction aggregates.

The CDW recycling has the potential to become an industry with financial growth expectations and could generate beneficial economic activities while reducing the environmental footprint. To obtain a quality product, the waste management and production of the CDW has to be appropriate and detailed, complying with the existing local regulations and applying the proper treatment to extend the service life of these materials. 

Regarding the incorporation of CDW to asphaltic mixes, the literature is scarce, with few examples of these materials used as petrous aggregates; while the majority of the uses are related to the production of hydraulic concretes Portland cement based. This research was conducted to determine if the incorporation of these materials could be feasible for the construction of roads. Other authors have experimented with CDW and found profitable uses which contribute to a sustainable development [7]. There were some works performed with mixtures of asphalt emissions and CDW, finding that the percentage of recycled aggregates can be up to 100% of the volume, provided that the humidity is controlled [7,8]. Other works developed new mixtures with CDW according to the Marshall methodology requirements, with substitutions up to 30% of the mineral aggregates, and achieving high mechanical resistances. Furthermore, the performance and susceptibility against moisture can be improved with the incorporation of CDW [9]. Perez et. al. published in 2006 a study of characterization of asphaltic mixtures with CDW, concluding that it is necessary to expand the research and continue with the critical analyses of these materials [10]. Almeida et. al. (2017) utilized micronized polyethylene terephthalate as an additive in asphaltic mixes, conducting two designs with the SUPERPAVE methodology [11]. On the other hand, Polaczyk et. al. (2021) carried out two designs of asphaltic mixes composed by calcareous aggregates and PG 64-22 binder; they validated the locking point concept to avoid the standard N design from the Superpave methodology, with the application of performance tests and the evaluation of the aggregate influence in the fatigue cracking [12].

### Concrete Construction and Demolition Waste

Recycled concrete is a product derived from the demolished and collapsed buildings, roads or other infrastructures which have ended their lifecycle and which core composition is made of concrete material. These products are named “Construction and Demolition Waste” (CDW), which after an appropriate management and quality control can be reintegrated to building processes as a substitute of the traditional petrous aggregates.

In recent research works, the indiscriminate exploitation of natural resources has been identified as one of the main causes of the environmental deterioration of the planet. These lines of research focus on the proposal and implementation of reuse and recycle strategies for mainly asphaltic mixtures. In the year 2013, the SEMARNAT (Secretariat of Environment and Natural Resources) published in Mexico a new legislation which compels the construction industry to implement waste management plans for the CDW, according to the regulation NOM-161-SEMARNAT-2011 [13], which stablishes the principal values and objectives, promoting the reuse, recycling and disposal of the residues.

It is important to know that the construction wastes are classified as special handling wastes; therefore, it is an obligation to properly dispose the management to recycle or reuse them. Recently, these strategies have spread to the emerging countries as a way to make them relevant while impacting positively in the environment. This tendency, so-called circular economy, revolves around the elimination of residues and the continuous use of the resources. The justification of implementing recycled hydraulic concrete aggregates lies in the contribution to the environmental preservation, the conservation of the natural resources, the non-exploitation of the natural quarries, the costs reduction and the rational use of energy [14].

In Mexico, 30 million tonnes of CDW are generated annually [4,15]. The majority of the residues are brought to landfills and clandestine dumps, generating environmental and visual pollution. These waste materials could be recycled with the adequate treatment [16]. Countries like Netherlands or Belgium have reached recycling rates higher than 75%, thanks to the promotion and awareness of the problem [17,18]. This fact is due to the scarcity of natural aggregates and spaces for the location of landfills. One of the strategies to achieve these elevated recycling rates has been the increase of the dumping taxes or the partial or total prohibition in certain countries like Netherlands or Denmark. Other countries like United Kingdom or Austria are following these directions, and their results have been growing around the 40% volume recycled. In the case of Mexico, small volumes of CDW are recycled, and more than the 85% of the material is sent to the landfills [19,20].

The construction industry and the operation and maintenance of buildings around the world generate the 65.2% of the total electrical energy consumption, a 36% of the global primary energy. They also generate the 50% of the Greenhouse gas emissions and 136 million of solid wastes per day (approximately 1.3 kg/person/day), while the 90% of these residues could be recycled in its entirety [21]. It is estimated that in Europe, the construction industry generates around the 25–30% of the total solid wastes; with a proper handling, Islam et al. calculated that in 2020, the 70% of these residues could be reused, recycled and reconverted [22]. A significant proportion of the CDW are rests from concrete and aggregates result of demolitions; for instance, in Malaysia, more than the CDW consist of concrete and aggregates [23]. For each housing square meter, two tons of raw material are required [24]; therefore, the energy consumption needed for the materials which constitute one dwelling would amount to approximately one third of the total energy consumption of one family over 50 years. Moreover, the CDW generation exceeds the annual ton per inhabitant.

The main objective of this research work is to analyse the use of CDW as a feasible alternative to diminish the proportion of natural aggregates without worsening the quality of the asphaltic mixtures. We have to consider the technological aspects, the economic impact, the proposal for the materials employed, and the environmental contribution. Considering all these parameters, it will be possible to incorporate CDW in roadway constructions and flexible pavements.

The methodology selected for the asphaltic mixtures design is the Marshall. The methodology stands out for its versatility and adaptability, as well as its economic application; for these reasons, it is the most employed methodology at national and international level. Regarding Mexico and its estimated 174,779 km paved roadways, approximately 90% of them were determined with the Marshall methodology, considering that they are secondary roads or feeding highways, which have medium or low transit. These roadways do not require the same quality as the main infrastructures which need a special design and more rigorous mixtures with expensive and new technologies.

On the other hand, the official methodology according to the Mexican Secretariat of Communications and Transportation, SCT, is, in fact, the Marshall methodology, considering that it is the most used in all the country for the design of asphaltic mixtures with dense granulometries. Consequently, the current use of the Marshall methodology has been researched and studied in Mexico with the publication of technical issues like the Technical Publication N°. 271 from the IMT (Mexican Institute of Transportation), where the Marshall methodology and the Superpave design are compared, and the results show similarities and a good reliability [25].

## 2. Materials and Methods

### 2.1. Materials’ Collection

The experimentation was performed with materials and aggregates available in the region of Morelia in Michoacan, Mexico, specifically, from the quarry “La Roka”, which processes materials like gravels and sands as a product of the crushing of the near stone quarries. We will refer to these from this point on as Recycled Concrete Aggregates (RCA).

**Figure 1:**
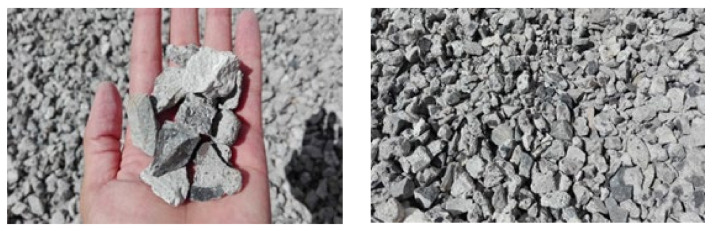
Recycled concrete aggregate (RCA).

The RCA were obtained from the crushing of concrete from an old building constructed in the 70s of the twentieth century, with nominal size of ¾” and a maximum particle size of 4.75 mm. The material is shown in Figure 1.

Once obtained the RCA, the waste material was subdued to a selection process to avoid polluting agents in the concrete. After the proper management and treatment of the CDW, we obtained a concrete waste material with lesser quantities of other elements which could contaminate the whole, like ceramic bricks, gypsum or mortars. Finally, the material was ground and cleaned to obtain the ultimate aggregate.

The material was collected and crushed in appropriate sizes for the experimentation. In this case study, the maximum size preferred was ¾” or 19 mm, which is commonly used for asphalt layers from 5 to 10 cm thickness. The asphalt employed in this research proceeds from the “Ing. Antonio M. Amor” refinery in Salamanca, Guanajuato, Mexico.

### 2.2. Materials’ Characterization

The experimentation was fulfilled in two phases:

First phase: Study and collection of the physico-mechanical properties of the NA and RCA, and also the asphalt. The Marshall methodology was followed for the design of the asphaltic mixture.

Characterization of coarse aggregates (NA and RCA):

Characterization of fine aggregates:

Dry compacted unit weight in fine aggregate by ASTM C29/C29M-17a [26], loose unit weight in fine aggregate by ASTM C29/C29M-17a [26], relative density ASTM C128-15 [27], absorption and surface moisture content by ASTM C128-15 [27], sand equivalent value of fine aggregates by ASTM D2419-14 [28], mechanical size analysis by ASTM D5444-15 [29]. 

Characterization of the asphalt:

Ductility by ASTM D 4402-15 [30], penetration test of bituminous materials by ASTM D5/D5M-2 [31], flash point of cutback asphalt by ASTM D3143/D3143M-19 [32], softening point of bitumen by ASTM D36-14 [33], relative density of asphalt by ASTM D71- 94 (2019) [34], viscosity determination of asphalt by ASTM D 4402-15 [30].

Second phase: coarse and fine materials composition for the asphaltic mixture with substitution of the NA by RCA (10, 20 and 30 percentages), for the optimum asphalt content.

Design of the asphaltic mixtures with RCA aggregates: The control mixture, as well as the other substitutions for petrous aggregates, was designed for a vehicular traffic between one million and 10 million ESAL’s (ESAL: the acronym of Equivalent Simple Axis Load and prediction of the quantity of load axis. The reference axle load is an 18,000-lb single axle with dual tires), according to the requirements of the Mexican regulations of the Secretariat of Communications and Transportation (SCT) and in accordance with the bituminous mixtures design using Marshall apparatus.

The following mixtures and dosages were designed according to the Marshall methodology: (1) 100% NA; (2) 90% NA, 10% RCA; (3) 80% NA, 20% RCA; (4) 70% NA, 30% RCA. For each mixture design, there were determined:

Granulometry: The granulometric curves of the aggregates were determined for each mixture design and compared with the requirements for asphaltic mixtures of the SCT and AASHTO T 2793 standards, with a nominal size of ¾” [35,36].

Minimum asphalt content: There were elaborated specimens of 101.6 millimetres in diameter and 64 millimetres of height to determine the minimum asphalt content. First, the approximate specific surface area of the petrous aggregates was determined with the granulometry and particle size; then, the specific surface area was multiplied by the asphaltic index to obtain the minimum asphalt content.

Volumetric parameters: The volumetric properties of the asphalt mixes are essential for an adequate design with optimum asphalt content. The main parameters are: air voids (AV), voids in the mixture (VM), voids in the mineral aggregate (VMA), voids filled by asphalt (VFA), and effective asphalt content (EAC). These parameters give an indication of the workability of the designed asphalt mix.

Optimum design of the asphalt: With the minimum asphalt content (3.7%), and according to the professional practice, it is recommended to increase to at least one percent. The experimentation initiated with 5% asphalt regarding the weight of the aggregates, also including the 5.5%, 6%, 6.5% and 7%. A total of 6 specimens were elaborated for 5, 5.5 and 7 percentages, and 8 specimens for the 6 and 6.5 percentages to increase the precision of the results. The specimens were tested to determine properties, such as stability, flow test, voids in the asphaltic mixture and voids filled by asphalt, and compare them with the regulations regarding the optimum asphalt content.

Frictional detachment of the mixture analysis: The percentage of the loss of adhesion in the uncompact asphalt-coated aggregates for the 70%NA/30% RCA mixture was obtained by submerging the specimens in boiling water during 15 min according to the standard ASTM D3625 [37].

**Figure 2:**
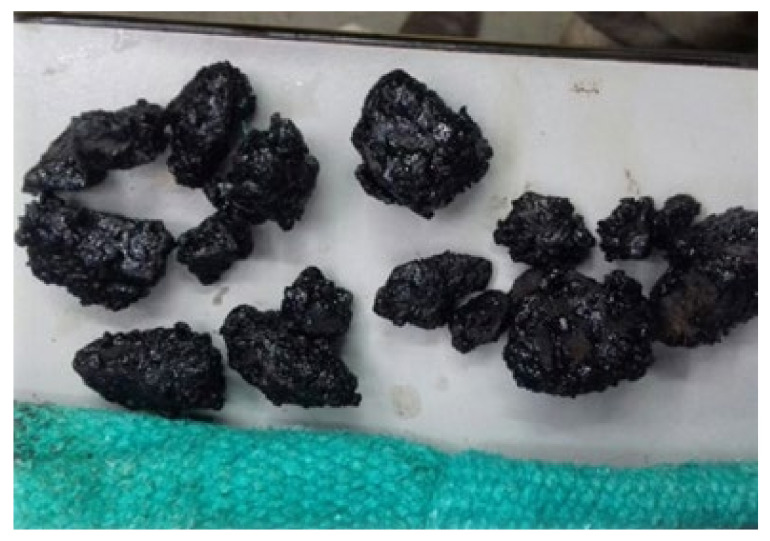
Detachment of the RCA asphaltic mixture.

As it can be seen in Figure 2, the detachment of the asphaltic aggregate layer is not really profound and does not represent any risk to the durability of the mixture. Nevertheless, it is necessary to mention that the RCA addition increases the detachment of the material.

Volumetric and gravimetric analyses of the compacted asphaltic mixture: With this analysis, for each design, the asphalt usage as well as the aggregate material dose per cubic meter of the mixture can be found.

There were 38 specimens elaborated for each asphalt mix, to determine the optimum content, with a total of 152 samples: 100% NA; 90% NA and 10% RCA; 80% NA and 20% RCA; and 70% NA and 30% RCA. Once the optimum content was established, the corresponding analyses set by Mexican regulation SCT or the corresponding ones by ASTM were performed, to determine the quality of the asphalt mixes.

## 3. Results and Discussion

The results of the different mixture designs were compared to the control sample specimens with 100% of NA and an optimum asphalt content of 6.66%; 90% NA and 10% RCA with optimum asphalt content of 6.70%; 80% NA and 20% RCA with optimum asphalt content of 6.84%; and 70% NA and 30% RCA with optimum asphalt content of 6.88%. The mixtures were designed to withstand a transit greater than one million and up to 10 million ESAL’S, complying with the requirements of the SCT regulation and the Marshall apparatus design methodology.

### 3.1. Characterization of Asphaltic Cement and Aggregates

The results of the physical and mechanical properties of the NA and RCA can be seen in Table 1, as well as the necessary tests to elaborate the asphaltic mixtures. 

### 3.2. Characterization of Petrous Aggregates Mixtures with Different RCA Percentage Substitutions

In addition to the trials shown in Table 1, the mixtures of petrous aggregates with different RCA percentages were tested by other means. Down below, the results of the test are shown. 

**Figure 3:**
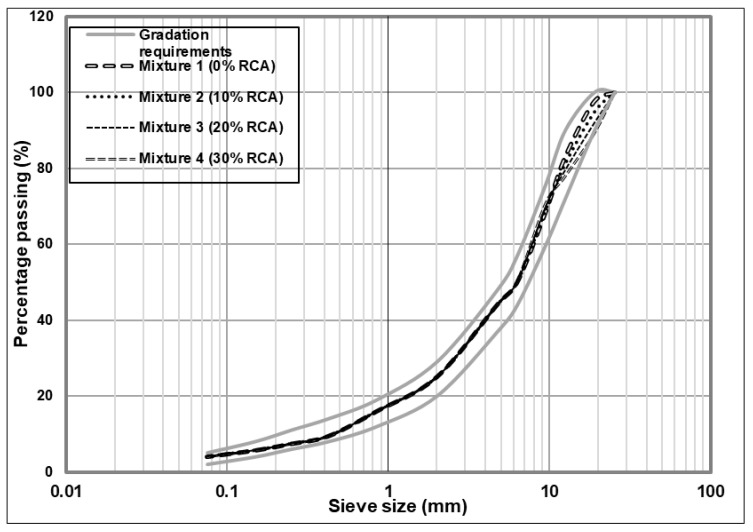
Granulometric curve of the petrous mixtures.

Figure 3 displays the distribution of the particle size for each one of the mixtures elaborated. It can be seen how all of them are placed between the limits recommended by the standards previously cited.

**Figure 4:**
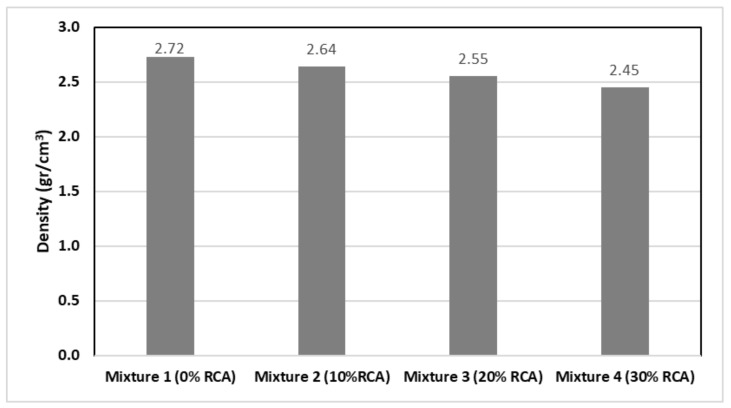
Density of the mixtures designed.

Figure 4 shows the density of the designed mixtures, by means of the Mexico Corps of Engineers methodology. The control sample, without the incorporation of RCA, presents the higher density value among all mixtures while the sample with 30% of RCA substitution has the lower value. It can be proven that the higher content of RCA in the mixture entails a lower density.

**Figure 5:**
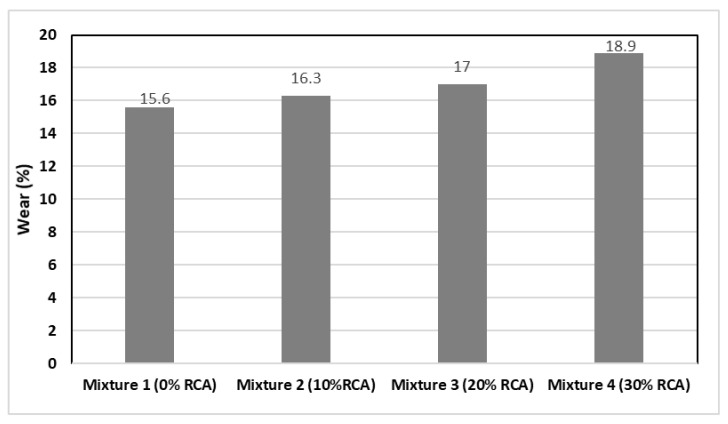
Resistance to degradation by abrasion and impact of the designed mixtures.

On account of the greater porosity and lower density of the mixture with 30% RCA substitution, the specimens with greater content of RCA also present a worst resistance to degradation by abrasion and impact as shown in Figure 5. Nevertheless, all the samples remain below the 30% maximum value stated by the ASTM C131/C131M-20 performed with the Los Angeles Machine [41]. Regarding the Los Angeles Resistance to Degradation by Abrasion test, the N-CMT-4-04/08 stipulates that when the expected traffic (ΣL) is less or equal to 1 million ESAL’s, the quality requirements entail a maximum percentage of 35% for high density. In discontinuous granulometry mixtures, the maximum percentage is 25%, and for the rest of cases, the open granulometries and any ΣL, it is 30%. In the present research, the values obtained had an average value of 15% (See Figure 4), which comply with the national regulations. This translates into a good behaviour and performance of the mixtures, avoiding the presence of bumps and the fast degradation of the asphalt layers.

**Figure 6:**
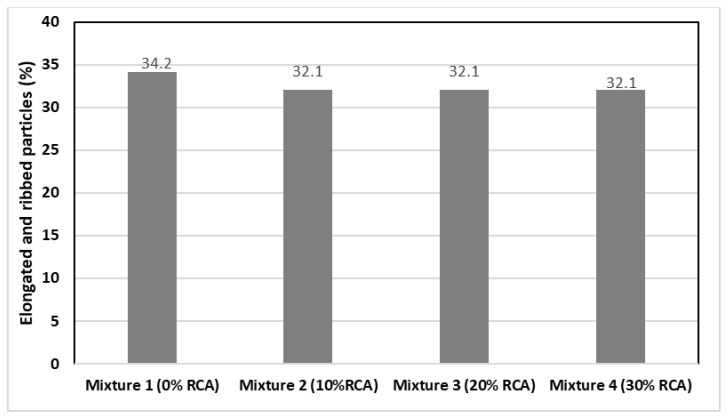
Percentage of Flat and Elongated Particles of the fine aggregates.

According to Figure 6, the control sample shows greater content of flat and elongated particles in contrast with the rest of the mixtures, which is expected as the RCA material goes through a trituration process which standardizes the aggregate form and size.

### 3.3. Design of Asphaltic Mixtures with the Incorporation of RCA as Petrous Aggregates Using the Marshall Methodology

In Table 2, the properties of each one of the designed asphaltic mixtures with different RCA percentages are shown, as well as the admission in the limits designated by the national regulations (according to the SCT), expecting a vehicular traffic between one million and 10 million ESAL’s.

Samples were elaborated for the designed mixtures with an average of 8 specimens for each percentage of aggregates (100% NA; 90% NA and 10% RCA; 80% NA and 20% RCA; 70% NA and 30% RCA). The specimens were compressed with 75 hits per side at a temperature of 160 °C.

**Figure 7:**
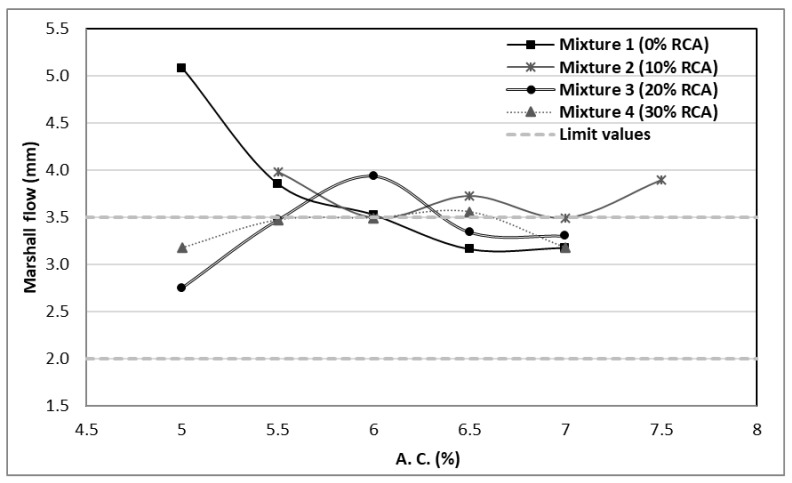
Flow values of the designed mixtures.

The flow value reflects the capability of the mixture to not suffer great deformation under repeated loads and stresses. The SCT sets the flow values allowed for asphaltic mixtures between 2 and 3.5 mm suitable for its use in roads with transits superior to one million ESAL’s. In Figure 7, it can be seen that the 6.5% and 7% asphalt contents with almost all the substitutions are within the range stablished by the SCT, except for the 6.5% asphalt and 10% RCA which fall over the 3.5 mm limit. 

**Figure 8:**
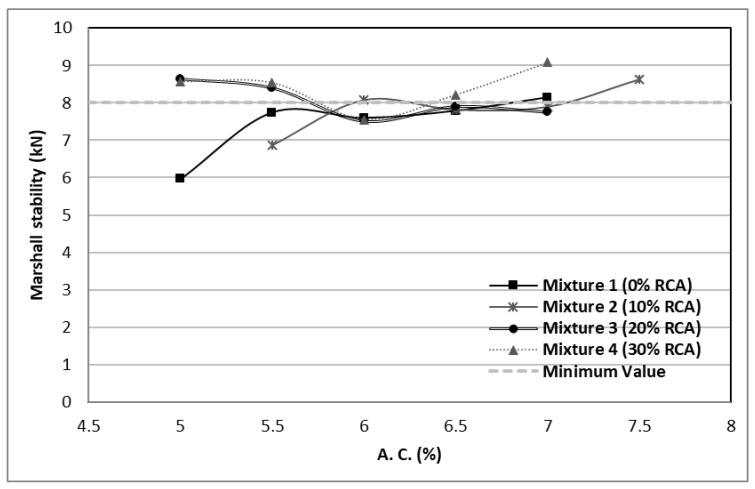
Stability values of the designed mixtures.

The stability of a mixture indicates its capability to resist the failure. Figure 8 enables to see that all the mixtures comply with the SCT regulations for road transit between one and ten million ESAL’s with at least one of the asphaltic cement contents. The minimum stability accepted for these asphaltic mixtures must be 8.16 kN. The mixture with higher values was the 7% cement and 30% RCA, which also obtained a density lower than the required by the standards (see Figure 3). 

It can be noted that decreased density caused by the increase of the RCA content does not affect the mechanical behaviour of the mixtures; conversely, it improves the performance of the product, especially for the 10% and 30% RCA percentages. In other research works, with a total substitution (100%) of the petrous aggregates by RCA, the mechanical values have also been very satisfactory [46].

**Figure 9:**
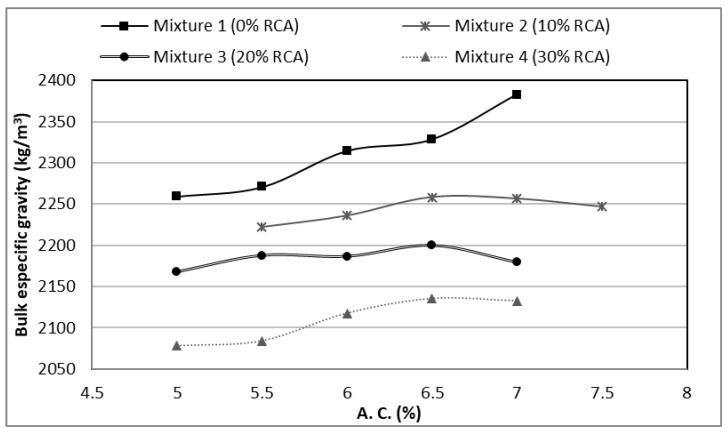
Specific gravity (bulk density) of the designed mixtures.

In Figure 9, it can be seen the specific gravity or bulk density of all the specimens. The control mixture (with 100% NA) presents better values than the substitutions; with a decrease in the density proportional to the increase of the RCA content. Figure 9 can be correlated with Figure 3, due to the lower density values obtained for the partial substitutions of the mixtures. This test has not confidence limits by the regulations. 

**Figure 10:**
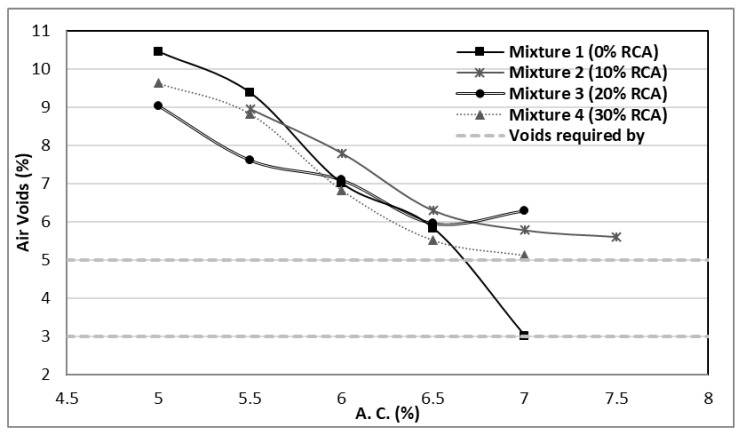
Air voids percentages of the designed mixtures.

In Figure 10, the percentage of air voids contained in the different mixtures are shown. It can be observed that the greater amount incorporated of RCA, the greater asphalt content needs to be added to fill the air voids to comply with the regulations. The standards require a percentage between 3% and 5%. The recycled concrete aggregates present a higher porosity; therefore, the mixtures will have more empty spaces in their structure, and consequently, more asphalt will be required to fill the voids. It is important to find a balance between this extra asphalt percentage and the stability and flow of the mixtures.

**Figure 11:**
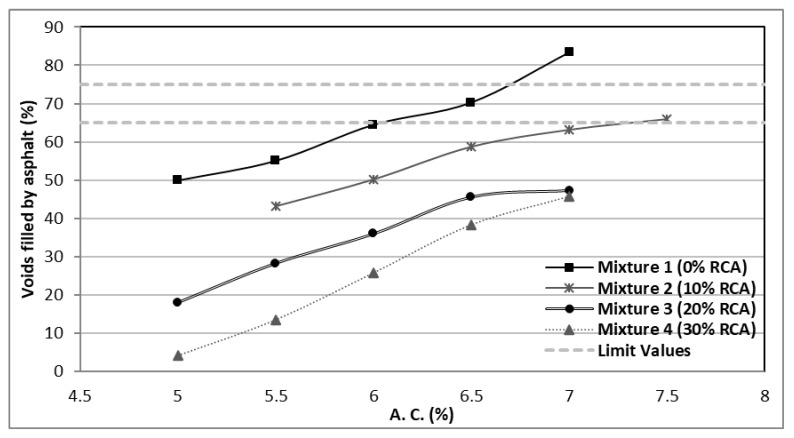
Voids percentage filled by asphalt in the mixtures.

In Figure 11, the percentage of voids filled by asphalt in terms of the asphalt content of the mixtures is shown. It can be observed that with a greater percentage of RCA, the voids filled by asphalt tend to decrease. This percentage of Voids Filled by Asphalt (VFA) indicates the optimum asphalt content to produce a durable mixture or, conversely, the more unreliable combinations.

The acceptable VFA range depends on the vehicular traffic levels. Higher traffic levels demand lesser VFA percentages, on account of the priority to elevate the stability and mechanical resistance. In Figure 11, it can be observed that the VFA for the RCA mixtures is lower than the control mixture; therefore, the designed products could be optimal for high transit roads.

**Figure 12:**
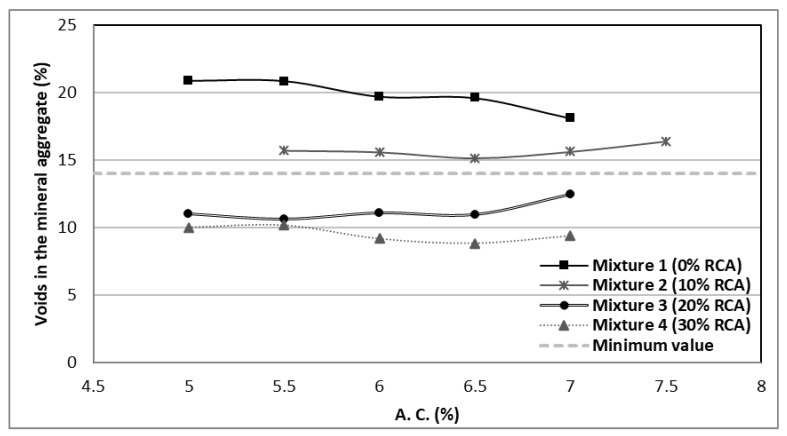
Natural aggregates voids percentage in the mixtures.

In Figure 12, the effect of the voids of the mineral aggregates (VMA) can be noted, which is the intergranular space occupied by the asphalt and the air inside a compacted asphaltic mixture, and expressed in a percentage of the total volume. 

The VMA illustrates the available area to settle the effective asphalt volume and the necessary air volume for the mixture. The minimum required percentages of VMA are essential to secure the adequate asphalt film thickness and ensure the durability. As it is observed in Figure 12, the better results correspond to the 10% and 20% RCA substitutions, as they fall closer to the values required in the regulations.

**Figure 13:**
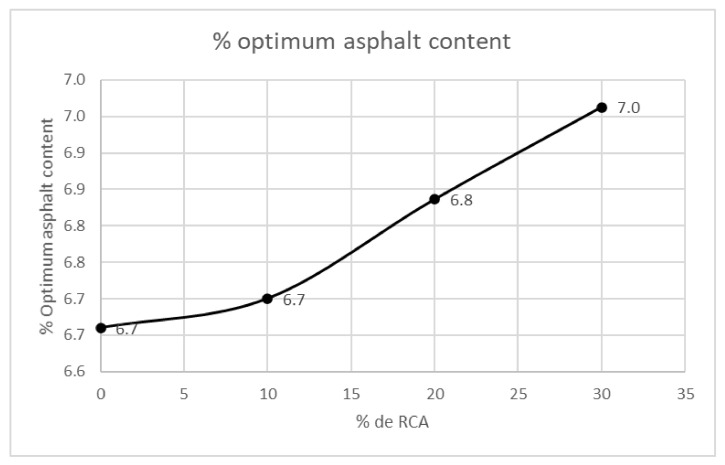
Optimum asphalt content regarding the RCA content of the designed mixtures.

In Figure 13, the optimum percentage of asphalt is represented as a function of the RCA content of the mixtures. The increase of the RCA provokes major asphalt content due to the greater porosity of the recycled concrete aggregates; consequently, more asphalt is required to fill the voids. This performance displayed in Figure 13 can be directly correlated with Figure 10, where the greater content of RCA causes a bigger amount of asphaltic cement for the mixtures.

**Figure 14:**
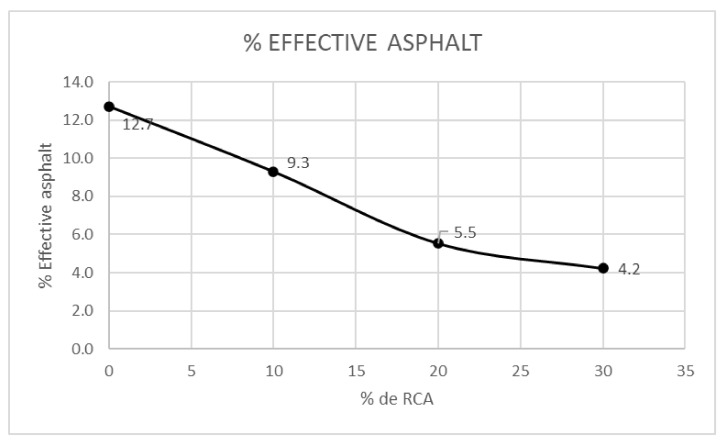
Effective asphalt by RCA content utilized.

In Figure 14, the percentage of effective asphalt over the total volume is shown. The effective asphalt refers to the quantity of asphalt which remains in the superficial area of the aggregate, meaning it has not been absorbed. It can be observed that the increase of RCA content tends to decrease the effective asphalt, in contrast to the absorbed asphalt. Figure 14 is directly related with Figure 11, which corresponds to the VFA, and it is possible to state that while the mixture complies with the parameters of Figure 10, the effective asphalt content will be acceptable. 

### 3.4. Correlation between the Asphaltic Mixtures Performance and the RCA Content

In this section, the RCA contents of the asphaltic mixtures, the optimum content of asphaltic cement and the different parameters analysed by the Marshall methodology will be compared. The objective is to determine the impact of the RCA incorporation in the asphaltic mixtures.

**Figure 15:**
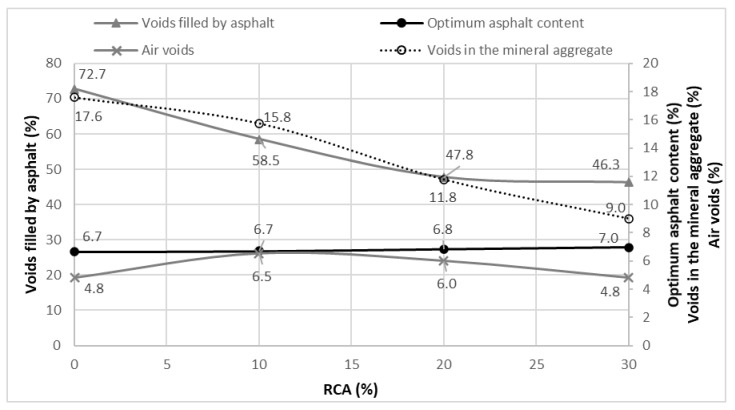
Correlation of VFA versus optimum asphalt content, VMA and air voids.

In Figure 15, the VFA are compared with the VMA, air voids percentage and optimum asphalt content. It is noted that the greater RCA contents provoke lesser voids in the mixture, lesser voids filled by asphalt and an increase in the optimum asphalt content. The RCA present a low amount of flat and elongated particles (see Figure 6); therefore, as the RCA content grows, the VMA and VFA percentages tend to decrease, being the last two directly correlated. Regarding the optimum asphalt content, it can be noted that its increase is not too high in comparison with the RCA addition. 

**Figure 16:**
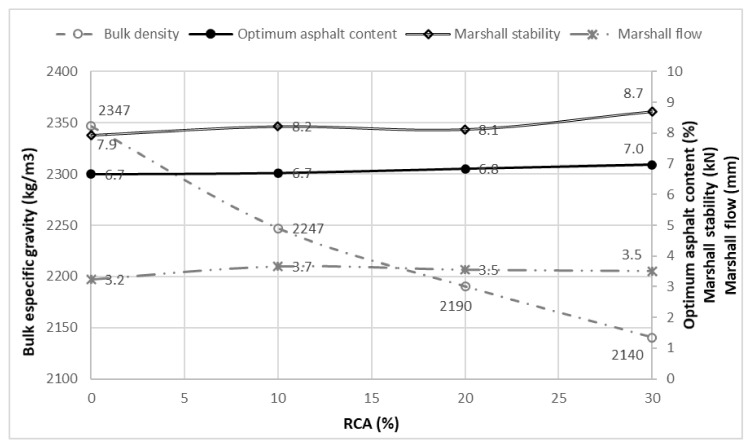
Correlation of bulk density versus optimum asphalt content, Marshall stability and Marshall flow.

Figure 16 shows the behaviour of the mixture regarding the optimum asphalt content, the stability and flow in comparison with the RCA content and the bulk specific gravity. While the optimum asphalt content, the stability and the flow experiment a slight growth as the RCA content increases, the bulk specific gravity tends to decline, due to the lower density of the RCA aggregates in contrast to the conventional natural aggregates.

It is important to note that the density reduction of the RCA mixtures did not decrease the mechanical properties (see Figure 8 and Figure 9); the reason could be the interfacial transition area between the recycled aggregate and the asphaltic cement [47], which is determining for the mechanical behaviour of the mixtures.

**Figure 17:**
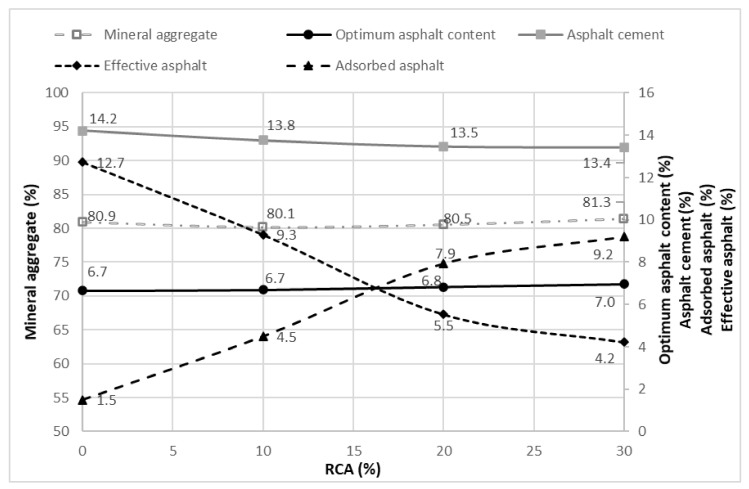
Correlation of the mineral aggregate percentages versus the optimum asphalt content, asphaltic cement, absorbed asphalt and effective asphalt.

Figure 17 shows that when the content of RCA increases, the absorbed asphalt percentage does the same while the percentages of asphaltic cement and effective cement decrease. This situation can be explained by the greater porosity of the RCA materials (see Figure 4) and the major absorption they present (see Table 1). Other research works have obtained similar results with a greater absorption of the asphalt content in the designed mixtures [48]. An increase in the mineral aggregate amount can also be noted as the RCA content tends to grow.

In Table 3, it can be seen how the 70%NA/30%RCA presents the better stability in comparison with the control sample.

**Figure 18:**
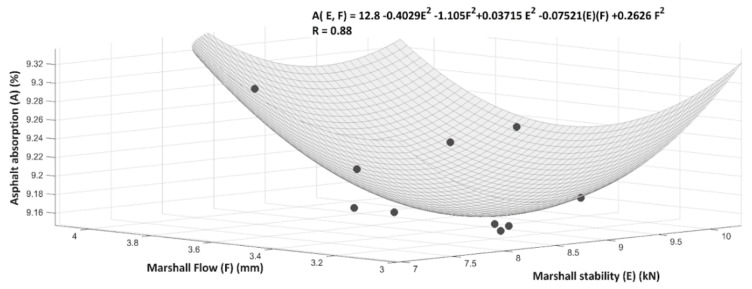
Correlation between Marshall stability, Marshall flow and percentage of asphalt absorption (Mixture with 30% RCA).

Figure 18 shows the correlation between Marshall stability, Marshall flow and the percentage of asphalt absorption of the mixture with 30% RCA aggregate by means of a polynomial surface and a mathematic model. The correlation coefficient R has a value of 0.88, which means a good performance of the model designed.

## 4. Conclusions

One of the main revelations of the research is that an increase of the RCA aggregates also increases the absorbed asphalt in the mixture, while effective asphalt content diminishes due to the higher porosity of the RCA material. Furthermore, the VMA and VFA decrease their values below the requirements of the SCT regulations.

The designed mixtures present good flow and stability properties assessed by the Marshall methodology, with a similar behaviour than the conventional mixtures. It is important to not forget about the durability, because the big amount of absorbed asphalt could end its lifecycle if the maintenance is not well done. On the other hand, this high asphalt absorption could be the cause of the great mechanical performance of the designed mixtures, even with the 30% RCA substitution, as it is shown in Table 3.

On the whole, it can be observed that a greater content of RCA generates an increase in the absorbed asphalt and stability, while the percentages of effective asphalt and specific gravity tend to diminish. The other properties remain in the standard values with slight increasing trends. The research works of Sanchez-Cotte et al. provided similar results, warning that the origin and properties of the utilized RCA are very important, since they can generate very different results during the experimentation [49]. The suitable processing, selection and management of the RCA will be reflected in greater quality asphaltic mixtures.

On the other hand, the high content of air voids caused by the designed mixtures will enhance the permeability but decrease the modulus and the durability [50]. It also has been demonstrated that the velocity of the curing of an asphaltic layer is directly related with the total air voids of the mixture; furthermore, the interconnectivity of the voids could affect the water transmission and consequently provoke the failure of the pavement due to the resistance loss in the base of the layer. According to the Table 2, it can be determined how the 30%RCA mixture complies with the air voids percentage regulated. In the same vein, the performance of the Los Angeles Resistance to Degradation by Abrasion test verified the fulfilment of the RCA as aggregates and also helped to corroborate the durability of the mixtures indirectly.

It was also observed that the detachment of the aggregate asphaltic layer was not really substantial, and therefore, the durability of the designed mixture is not threatened. Nevertheless, the increase of RCA percentage involves a slight increase in the detachment of the layer, which should not be ignored. The research work should continue with durability analysis to verify the lifespan of the mixtures with tests like the stablished in the standard AASHTO T-321: “Standard Method of Test for Determining the Fatigue Life of Compacted Asphalt Mixtures Subjected to Repeated Flexural Bending” [51].

Nonetheless, it is necessary to note the potential disadvantages of using RCA as additions in asphaltic mixtures: first, the absorption coefficient of the RCA is slightly superior compared with the NA. Another one is the possible contamination of the material due to the presence of other substances, due to the extensive management to process the RCA. Lastly, the behaviour and performance of the mixtures could vary significantly according to the origin and quality of the recycled aggregates.

The employment of RCA inside asphaltic mixtures helps with the subsequent reuse of the materials, increasing the lifecycle of hydraulic concrete, reducing the solid waste volumes, diminishing the exploitation of non-renewable minerals and lowering the environmental emissions.

The better mixture design proposed corresponds to the 70% NA and 30% RCA, on account of the great energy saving: the diminishing of petrous aggregates volume goes from 2200.16 to 2002.25 kg/m^3^, and the asphalt volume goes from 146.53 to 137.75 kg/m^3^ regarding the control asphaltic mixture.

## Figures and Tables

**Table 1:** Tests applied to the asphaltic cement and aggregates utilized for the mixtures.

Test	Standard	Material	Values Obtained	Suggested Values
Effective specific gravity	SCT-M-MMP-4-04-003/2 [38] ASTM C-127-15 [36]	NA	2.72 g/cm^3^	Doesn’t apply
		RCA	2.45 g/cm^3^	
Bulk specific gravity	SCT-M-MMP-4-04-003/2 [38] ASTM C-127-15 [39]	NA	2.67 g/cm^3^	2.40 g/cm^3^
		RCA	2.20 g/cm^3^	
Absorption	ASTM C-128-15 [27]	NA	1.68%	Doesn’t apply
		RCA	7.67%	
Los Angeles Resistance to Degradation by Abrasion	SCT-M-MMP-4-04-006/02 [40] ASTM C131/C131M-20 [41]	NA	15%	30% maximum
		RCA	18.9%	
Flat and Elongated Particles in Coarse Aggregate	SCT- M-MMP-4-04-005/08 [42] ASTM 4791-19 [43]	NA	34%	40% maximum
		RCA	33.5%	
Sand Equivalent Value of Fine Aggregates	SCT-M-MMP-4-05-004-16 [44] ASTM D2419-14 [28]	NA	66.3%	50% minimum
Density	ASTM D71-94 [34]	Asphalt	1.03 g/cm^3^	Doesn’t apply
Viscosity Determination of Asphalt at Elevated Temperatures	SCT-M-MMP-4-05-005-02 [45] ASTM D4402/D4402M-15 [30]	Asphalt	165–159 °C (mixed) 152–147 °C (compaction)	Doesn’t apply

**Table 2:** Results of the design of asphaltic mixtures.

Properties of the Mixtures	Control	10% RCA	20% RCA	30% RCA	SCT Values
Optimum asphalt content (%)	6.66	6.70	6.84	6.88	–
Specific gravity (kg/cm^3^)	2.35	2.25	2.19	2.14	–
Stability (Kg)	809	838	828	885	816 min
Voids (%)	4.80	6.53	6.00	5.00	3–5
Flow (mm)	3.24	3.65	3.55	3.50	2–3.5
Voids of the Mineral Aggregate (VMA) (%)	17.6	15.8	11.8	9.1	14 min
Voids Filled by Asphalt (VFA) %	72.7	58.5	47.8	44.5	65–75

**Table 3:** Descriptive statistics of the different mixtures.

Mixture	Test	Half	Standard Deviation	Variance
Mixture 10% RCA	Marshall stability (kN)	8.06	0.95	0.9
Mixture 20% RCA		8.01	0.64	0.41
Mixture 30% RCA		8.43	0.72	0.51
Mixture Testing		7.53	1.06	1.11
Mixture 10% RCA	Marshall flow (mm)	3.75	0.26	0.07
Mixture 20% RCA		3.32	0.42	0.18
Mixture 30% RCA		3.41	0.32	0.1
Mixture Testing		3.69	0.76	0.57

## Data Availability

Data is contained within the article.
